# Stairway Plot 2: demographic history inference with folded SNP frequency spectra

**DOI:** 10.1186/s13059-020-02196-9

**Published:** 2020-11-17

**Authors:** Xiaoming Liu, Yun-Xin Fu

**Affiliations:** 1 USF Genomics & College of Public Health, University of South Florida, Tampa, FL USA; 2 Department of Biostatistics and Data Science, School of Public Health, The University of Texas Health Science Center at Houston, Houston, TX USA

## Abstract

Inferring the demographic histories of populations has wide applications in population, ecological, and conservation genomics. We present Stairway Plot 2, a cross-platform program package for this task using SNP frequency spectra. It is based on a nonparametric method with the capability of handling folded SNP frequency spectra (that is, when the ancestral alleles of the SNPs are unknown) of thousands of samples produced with genotyping-by-sequencing technologies; therefore, it is particularly suitable for nonmodel organisms.

**Supplementary information:**

**Supplementary information** accompanies this paper at 10.1186/s13059-020-02196-9.

## Introduction

Demographic history is one of the most important forces shaping the polymorphic pattern of genomes. Conversely, DNA polymorphisms can be used to infer histories of population events, including, but not limited to, expansion, shrinking, bottleneck, migration, split, and admixture. In recent years, several methods have been developed to infer population size changes over time without the need for specifying parameters of the underlying population model [1–8], which are referred to as nonparametric or model-flexible methods. Among them, Stairway Plot [5, 9] (aka Stairway Plot 1) has proven applicable to relatively large samples (hundreds) using unphased sequence data produced by a wide range of sequencing technologies, such as low-depth sequencing [5] and RAD-seq [10], which makes it attractive to infer recent population histories of nonmodel organisms. However, as most of the methods mentioned above still require polarized SNP data for unfolded SFSs, i.e., the ancestral allele of each SNP needs to be known, which poses difficulties to its application to nonmodel organisms [11]. Here, we present Stairway Plot 2, which, compared to Stairway Plot 1, achieves significant improvement in terms of (1) the application to both folded and unfolded SFSs, (2) overfitting control, (3) speed, (4) support for masking out part of the SFSs, and (5) usage convenience.

## Results

**Fig. 1:**
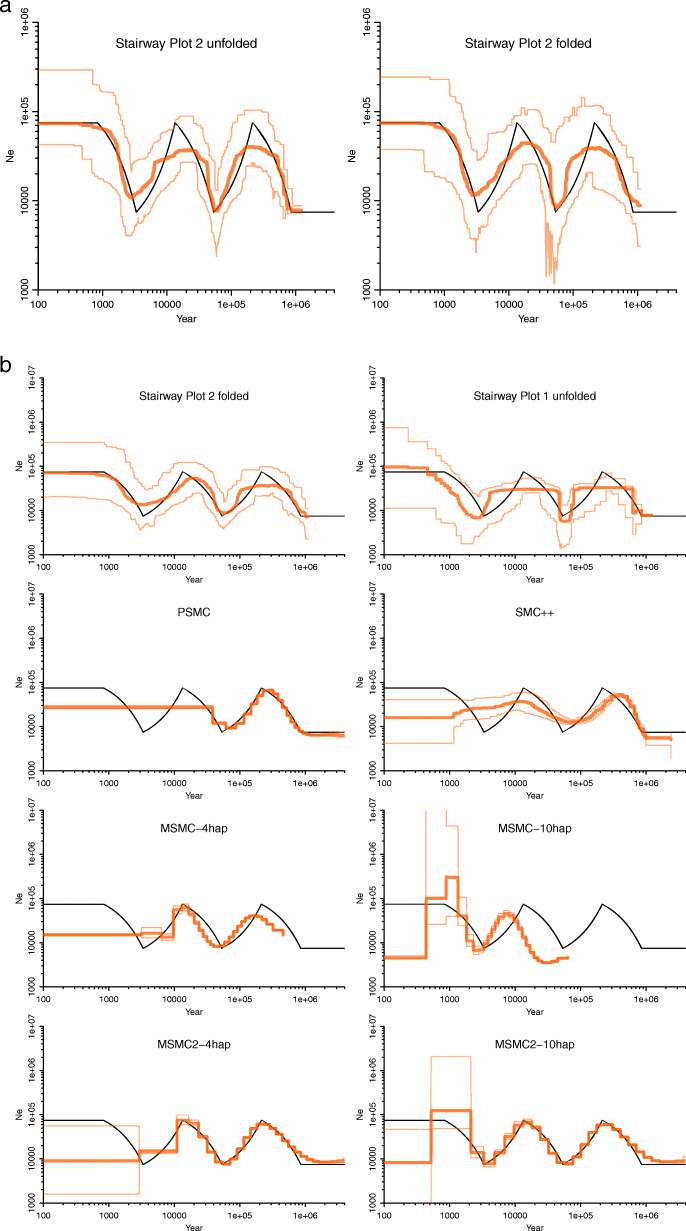
Comparison of demographic inferences with simulation. **a** Comparison of Stairway Plot 2 with folded or unfolded SFSs using the same average SFS from 200 simulations. **b** Comparison of Stairway Plot 2 with folded SFSs vs. Stairway Plot 1, PSMC, SMC++, MSMC, and MSMC2, using the same simulated sequences from 200 simulations assuming a zig-zag model [2]. Each simulation simulates 100 diploids with 10 chromosomes; each chromosome is 10 MB. Only one estimation for each simulated sample was used for Stairway Plot 1 and Stairway Plot 2. MSMC and MSMC2 group samples with every 4 haplotypes (4hap) or every 10 haplotypes (10hap). Black line: true model. Thick orange line: median of 200 estimations. Thin orange lines: 2.5% and 97.5% confidence limits for 200 estimations

Stairway Plot 2 can now be applied to both folded and unfolded SFSs and, therefore, no longer requires inferring the ancestral alleles as a prerequisite. For folded SFSs, the composite likelihood function is defined (see the “Methods” section). For the reason of the identifiability of the demographic model [12], the maximum number of epochs used in the underlying multi-epoch model [5, 13, 14] need to be equal to or smaller than the counts of the observed folded SNP type (i.e., *η* s), including the number of monomorphic sites. We compared the performance of Stairway Plot 2 using either unfolded SFSs or folded SFSs with the same single SFS and found that the final estimations, i.e., the median of the inference ensemble of subsampled SFSs (by default 200), are similar in general (Fig. 1a, Additional file 1: Fig. S1). In contrast, the variations (defined by the 95% confidence intervals of the inference ensemble) in ancient history inference for the folded SFSs can be wider than those in the unfolded SFSs due to loss of information. On the other hand, loss of information may help to mitigate model overfitting. Therefore, the impact of the loss of information can be complex and depends on the underlying demography. We can investigate the impact by comparing the mean squared error (MSE) of the estimations with folded or unfolded SFSs (Additional file 1: Fig. S2). For example, Additional file 1: Fig. S2A compared the MSE of 200 subsample estimations with either folded or unfolded SFSs used in Fig. 1a. For most of the history, especially for more ancient histories, the estimations with unfolded SFS have a similar or smaller MSE, while in some periods those with folded SFS have a smaller MSE. Please note that in the figures, we used log-scale for both the time (*x*-axis) and effective population size (*y*-axis), which emphasizes more recent histories and smaller population sizes.

Controlling overfitting is essential for demographic history inference because the overfitted model not only underperforms but may also suggest artificial fluctuations in the population size [6, 11, 15, 16]. Controlling overfitting is especially relevant for model-flexible methods, as they typically search a wider model space and involve more parameters than model-fixed methods, such as ∂a∂i [17]. Inspired by the random forests [18] method, Stairway Plot 2 controls overfitting by setting constraints on the parameters and model space. First, SFS bootstrapping is replaced by SFS subsampling [19]. A subsample (by default 2/3) of the observed sites is used to create an SFS training set and train the multi-epoch model, and the remaining sites are used to create an SFS testing set and test the goodness of fit of the trained model. Second, the number of “breakpoints”, which define the boundaries of each epoch, is further constrained. For a sample of *n* sequences, there are a total of *n* − 2 potential break points. By default, Stairway Plot 2 tests the goodness of fit of the trained models (with the ensemble of testing SFSs) using ¼, ½, ¾ or all of the *n* − 2 breakpoints, and the best-fit model is used for producing the final inference. Users have the option to add/use alternative numbers or fine-tune the numbers to find the optimal one that has the best goodness of fit for the testing SFSs.

To evaluate this new procedure, we compared the performance of Stairway Plot 2 with several other model-flexible methods, namely, Stairway Plot 1 [5], PSMC [1], MSMC [2], MSMC2 [8, 20], and SMC++ [4], using simulated sequences assuming several demographic models (Fig. 1b, Additional file 1: Fig. S3, S4). In the comparison, MSMC and MSMC2 used phased and polarized data, Stairway Plot 1 and SMC++ used unphased and polarized data, and Stairway Plot 2 and PSMC used unphased and unpolarized data. As Stairway Plot 1 and Stairway Plot 2 typically produce an ensemble of estimations, based on which the final estimation and confidence intervals are derived, while all other methods produce a single estimation for each simulated sample, to make the comparison fairer, only one estimation for each simulated sample was used for Stairway Plot 1 and Stairway Plot 2. For each demographic model, a sample of 100 diploids (200 haploids) was simulated for each simulation, and 200 independent simulations were conducted. For the extensions of the PSMC, we observed that 1) MSMC is not stable when using high haplotype size (hap = 10); 2) MSMC2 and SMC++ outperforms MSMC and PSMC as to estimating recent histories (Additional file 1: Fig. S3, S4). Stairway Plot 1 and Stairway Plot 2 better infer recent histories than PSMC, MSMC, SMC++, and MSMC2. Stairway Plot 2 also performs better than Stairway Plot 1, even though folded SFSs were used for Stairway Plot 2, while unfolded SFSs were used for Stairway Plot 1. The artificial bottlenecks sometimes produced by Stairway Plot 1 near the inference limit of ancient histories (e.g., in Additional file 1: Fig. S4b) were also well mitigated by Stairway Plot 2 (see Additional file 1: Fig. S4a). Stairway Plot 2 can also provide a more robust estimation of the inference variation (e.g., confidence intervals) compared to other methods, regarding the overlapping of the 2.5% to 97.5% inference range with the true models.

**Fig. 2:**
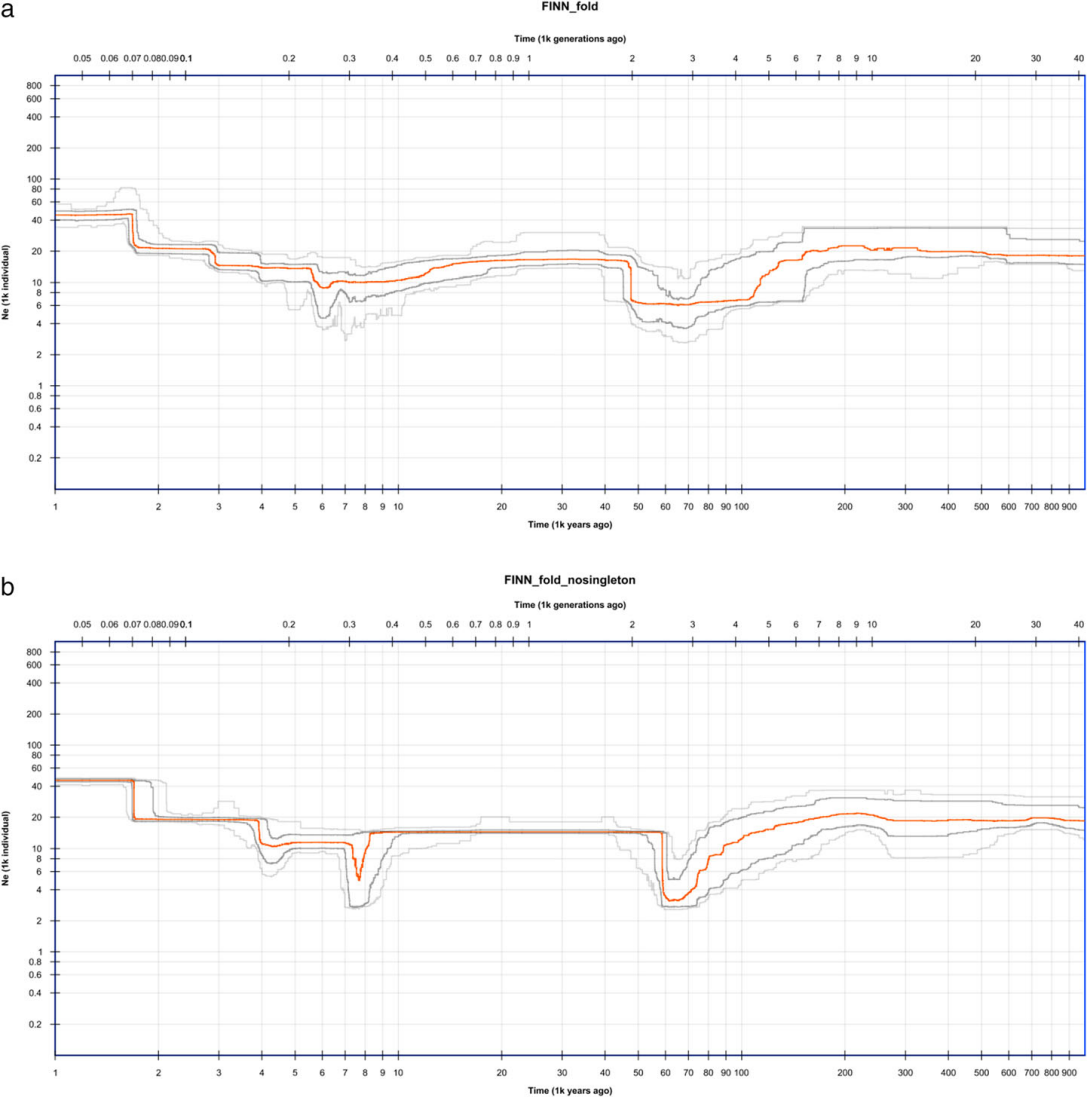
Inferred demographic history of the Finnish population based on 1747 individuals. **a** Stairway Plot 2 inference with folded SFSs. **b** Stairway Plot 2 inference with folded SFSs and masking singletons. Orange line: median of 200 inferences based on subsampling. Dark gray lines: 75% confidence interval of the inference. Light gray lines: 95% confidence interval of the inference

Java programs have also been rewritten for Stairway Plot 2 to improve efficiency. A speed increase of 10 × or more compared to Stairway Plot 1 was often achieved based on our simulation studies. For example, on a single thread of an Intel Xeon Gold 5122 CPU @ 3.60 GHz, the time required for Stairway Plot 1 to produce the results (unfolded, 200 subsample estimations) for Fig. 1b was 19,096 min. In contrast, only 900 min were required for Stairway Plot 2 (unfolded, 800 subsample estimations), that is, a 21-fold speed increase. With the same setting for producing results for Additional file 1: Fig. S3 and S4, Stairway Plot 1 required 15,839 and 9619 min, while Stairway Plot 2 required 704 and 540 min: a 22-fold and 18-fold speed increase, respectively, were achieved. With the faster speed, Stairway Plot 2 can handle a sample size of thousands of sequences given that an HPC cluster is available. To demonstrate its capability, we applied Stairway Plot 2 to the SFSs of 1747 Finnish individuals using 650M neutral SNPs from the Genome Aggregation Database (gnomAD) [21] (Fig. 2a). The result suggests a bottleneck between 40 and 200 thousand years ago (kya) based on the 95% confidence interval, likely due to out-of-Africa migration. It also suggests a recent 2-fold population growth approximately 2 kya and a shallow bottleneck between 4 and 10 kya with a bottom approximately 6 kya, which may be related to ancient migration events following the retreat of glaciation.

Stairway Plot 2 now officially supports masking out part of the SFSs, for example, singletons. Because calling singletons is often more complicated than calling SNPs with higher frequencies, inference with SFSs without singletons may help to identify inferred population events that are dominated by singleton information and, therefore, less reliable. We applied this technique to the Finnish data. We found that the bottlenecks 40–200 kya and 4–10 kya bottleneck and population growth ~ 2 kya are still supported, but the bottom of the 4–10 kya bottleneck shifts to 7–8 kya (Fig. 2b).

## Conclusions

In summary, Stairway Plot 2 is a significant improvement over Stairway Plot 1. By modeling folded SFSs and using an unsupervised learning strategy for model selection, it provides a more accurate inference of demographic histories. It is especially suitable for nonmodel organisms, as the challenging steps of phasing and SNP polarization are no longer needed. The software, along with its source codes and instruction, is freely available at https://github.com/xiaoming-liu/stairway-plot-v2.

## Methods

### Brief introduction of the Stairway Plot method

The flexible multi-epoch model used in the skyline plot method [13, 14] is implemented for the Stairway Plot, which divides time into a series of blocks with each block starting and ending at the exact time of a particular coalescent event in the sampled sequences. The population size is assumed to remain constant within each block and to be able to change from one block to the next. A maximum of *n* − 1 time blocks can be defined given a sample of *n* DNA sequences, with block *k* corresponding to the *k*-coalescent time. Those *n* − 1 time blocks can be approximated to any demographic history. The Stairway Plot estimates a series of *θ*_*k*_, *k* = 2, 3, …, *n*, maximizing the likelihood of the observed SFS. *θ*_*k*_ = 4*N*_*k*_*μ*, where *N*_*k*_ is the effective size of the population during time block *k*, and *μ* is the mutation rate per bp per generation. In practice, adjacent blocks of time can be fused into one block to reduce the parameters to be estimated. More details of the algorithm can be found in [5].

One of the major improvements for Stairway Plot 2 is removing the requirement of polarizing SNPs by modeling folded SFS, and better model selection by using an unsupervised learning strategy. The major challenges are (1) whether the Stairway Plot framework will work with half the number of observations with folded SFS and (2) whether the loss of information can be compensated by better model selection strategy. The results showed that the Stairway Plot framework works with folded SFS and performs well with the new model selection strategy. This is partially contributed to the ensemble step: although each individual estimation can be coarse (fewer epochs), the ensemble estimation can be smooth and accurate.

### Brief introduction to Stairway Plot 2

Let *t*_*k*_ be the *k*-coalescent time of a random sample of *n* sequences, *N*_*k*_ is the effective size of the population during *t*_*k*_, *θ*_*k*_ = 4*N*_*k*_*μ*, and *μ* is the mutation rate per bp per generation. *θ*_*k*_ s are estimated for each of the *B* (default is 200) sub-samples of the SFS instead of bootstrap samples as in the Stairway Plot 1 [5]. In Stairway Plot 2, the effective population size trajectory is calculated for each SFS sub-sample, that is ${N}_e^b(T)={\theta}_k^b/\left(4\mu \right)$ if ${T}_i^b<T\le {T}_{i-1}^b$, where ${\theta}_k^b$ is the *θ*_*k*_ estimation based on the sub-sample *b*, and ${T}_i^b=\sum \limits_{k=i}^n\frac{\theta_k^b}{k\left(k-1\right)},i=2,3,\dots, n.$

Then at each time point *T*, the median of a total of *B* estimates of the effective population size ${N}_e^b(T)$ is used as the final estimate of *N*_*e*_ at *T* [9].

### Composite likelihood of folded SFS

Composite likelihood of the observed SFS was calculated as:

$$ {L}_n={l}_n!\prod \limits_{i=0}^{n/2}\frac{p_i^{\eta_i}}{\eta_i!}, $$

where *n* is the sample size (number of haploids), *η*_*i*_ is the count of observed sites with a minor allele count of *i*, *p*_*i*_ is the frequency of *η*_*i*_ in the samples, and ${l}_n=\sum \limits_{i=0}^{n-1}{\eta}_i$. This likelihood is calculated for both the training purpose (with training data) and testing/evaluating purpose (with testing data).

### SFS subsampling

Let *l*_*n*_ be the total number of sites observed, as defined above, where *n* is the sample size (number of haploids). A number ${l}_n^{\prime }$ (by default ${l}_n^{\prime }=2/3{l}_n$) sites are randomly sampled from *l*_*n*_ sites and used as training data. The remaining ${l}_n-{l}_n^{\prime }$ sites are used as testing data. SFSs, either folded or unfolded, can be obtained by summing the SNPs of a given ancestral allele count (unfolded) or minor allele count (folded).

### Constraint on “breakpoints”

For a sample of size *n*, a maximum of *n −* 1 different *θ*s that can be estimated. In an ordered serial of *θ*_2_, *θ*_3_, …, *θ*_*n*_, “breakpoints” are inserted into the serial that separates the *θ*s into continuous groups. Any two consecutive *θ*s that are not separated by a breakpoint belong to the same group. The *θ* s within the same group have the same value, while those belonging to different groups may have different values. Therefore, there are *n* − 2 possible breakpoints that can be inserted. The actual number of breakpoints to be inserted into the serial is defined by the “blueprint” file. By default, four numbers approximately equal $\frac{1}{4}\times \left(n-2\right)$, $\frac{1}{2}\times \left(n-2\right)$, $\frac{3}{4}\times \left(n-2\right)$, and *n* − 2 are used. Given a number *m*, for each training SFS, from the full set of breakpoints (i.e., 1, 2, …, *n* − 2) *m* of them are randomly picked. The best grouping of *θ*s fitting the training SFS follows the same procedure described in the Stairway Plot 1 paper [5] with the constraint that the actual breakpoints must be chosen from the *m* break points.

### Determine the best number of “breakpoints”

For *m* breakpoints defined above, the best estimations of *θ* s are obtained for each training SFS using the procedure described in the Stairway Plot 1 paper [5]. Then, the likelihood of this set of *θ*s using the corresponding testing SFS is calculated and used as the measurement of goodness-of-fit of those *θ*s. The average goodness-of-fit of a total of *B* testing SFS is used for the overall goodness-of-fit of using *m* break points, *G*_*m*_. With a set of *m*, the best *m* is the one with the largest *G*_*m*_. In practice, considering the variation of *G*_*m*_, the best *m* is picked as the smallest number *m* that satisfies ${G}_m>{G}_{m^{\prime }}+1.92$ for all *m*^′^ < *m*, where *m* and *m*′ are both from the set of *m*.

### Simulation

SNP data were simulated using the ms [22] or MaCS [23] (Markovian Coalescent Simulator) programs. If not specified, all SNPs were simulated assuming a mutation rate (*μ*) of 1.2 × 10^−8^ per base pair per generation, a recombination rate of *ρ* = 1.2 × 10^−8^ per base pair per generation, and a generation time of 24 years. Simulation commands used for producing the data used in Additional file 1: Fig. S1 can be found in the Supplementary Note of the Stairway Plot 1 paper [5]. Other simulation commands are listed below.

zig-zag model: for /L %%i in (1, 1, 10) do (ms 200 200 -t 7156.0 -r 7156.0 10000000 -eN 0 5 -eG 0.000582262 1318.18 -eG 0.00232905 -329.546 -eG 0.00931619 82.3865 -eG 0.0372648 -20.5966 -eG 0.149059 5.14916 -eN 0.596236 0.5 >zig-zag-10M-%%i.out)

sharpCEU model: macs 200 30000000 -i 200 -t 0.0007156 -r 0.0007156 -eN 0.0 10.8300726663 -eN 0.00116452394261 1.08300726663 -eN 0.0174678591392 0.216601453326 -eN 0.0465809577045 1.08300726663 -eN 0.0873392956959 3.24902179989 -eN 0.232904788522 1.08300726663 2>/dev/null >sharpCEU.macs.out

sharpYRI model: macs 200 30000000 -i 200 -t 0.001 -r 0.001 -eN 0.0 8.25 -eN 0.0025 0.825 -eN 0.0416666666667 2.475 -eN 0.166666666667 0.825 2>/dev/null >sharpYRI.macs.out

### SFS of the Finnish individuals

The gnomAD project whole genomes sites and allele frequencies of the Finnish individuals were downloaded from http://gnomad.broadinstitute.org/downloads. A total of 650,351,035 likely neutral sites that are 50 kb away from any known coding genes yet within the 1000 Genomes Project phase 1 [24] strict mask were used for analyses [5].

### Parameters used in PSMC, SMC++, MSMC, MSMC2, Stairway Plot 1, and Stairway Plot 2

The PSMC estimations were conducted using the default parameters tuned for human populations: -N25 -t15 -r5 -p “4+25*2+4+6”. The composite likelihood with all individuals in the sample was used in PSMC. For SMC++, as suggested by its readme file, the composite likelihood using 10 distinguished individuals was used. The parameters “--regularization-penalty 5.0 --knots 16 --timepoints 35 100000” were used for SMC++ as suggested [6]. MSMC and MSMC2 used parameters “--skipAmbiguous”, and “-r 1” since we know the simulated recombination rate equals to the mutation rate. MSMC also used parameter “--fixedRecombination” as recommended by the authors [25]. For Stairway Plot 1 and 2, the default parameters were used.

## Supplementary information

**Additional file 1: Fig. S1-S4**.

**Additional file 2.** Review History.

## Data Availability

This study makes use of data generated by the gnomAD consortium [21]. Details of download links can be found in the “Methods” section. The Stairway Plot v2 software is freely available at Github [26]. Raw data supporting the results presented in the paper and the Additional file 1 can be obtained from Zenodo [27].
