# Author Correction: Stairway Plot 2: demographic history inference with folded SNP frequency spectra

**DOI:** 10.1186/s13059-020-02243-5

**Published:** 2020-12-27

**Authors:** Xiaoming Liu, Yun-Xin Fu

**Affiliations:** 1 USF Genomics & College of Public Health, University of South Florida, Tampa, FL USA; 2 Department of Biostatistics and Data Science, School of Public Health, The University of Texas Health Science Center at Houston, Houston, TX USA

**Correction to: Genome Biol (2020) 21:280**

**https://doi.org/10.1186/s13059-020-02196-9**

Following publication of the original paper [1], the authors reported an error.

After the heading “**Composite likelihood of folded SFS**”, it states “*p*_*i*_ is the frequency of *η*_*i*_ in the samples”. This sentence should read as “${p}_i=E\left({\eta}_i|{\theta}_2,\cdots, {\theta}_n\right)={\sum}_{k=2}^{n-i+1}\frac{\Gamma \left(n-i\right)\Gamma \left(n-k+1\right)}{\Gamma \left(n-i-k+2\right)\Gamma (n)}{\theta}_k+{\sum}_{k=2}^{i+1}\frac{\Gamma \left(n-i\right)\Gamma \left(n-k+1\right)}{\Gamma \left(n-i-k+2\right)\Gamma (n)}{\theta}_k$ is the expected frequency of *η*_*i*_ in the samples given *θ*_2_, ⋯, *θ*_*n*_”.
