# Supplementary material for: Stairway Plot 2: demographic history inference with folded SNP frequency spectra
Source: Genome Biol. 2020 Nov 17;21:280. doi: 10.1186/s13059-020-02196-9 (PMC7670622; doi:10.1186/s13059-020-02196-9)
Supplement: Supplementary file 1 — Additional file 1: Fig. S1-S4. [file 13059_2020_2196_MOESM1_ESM.docx]

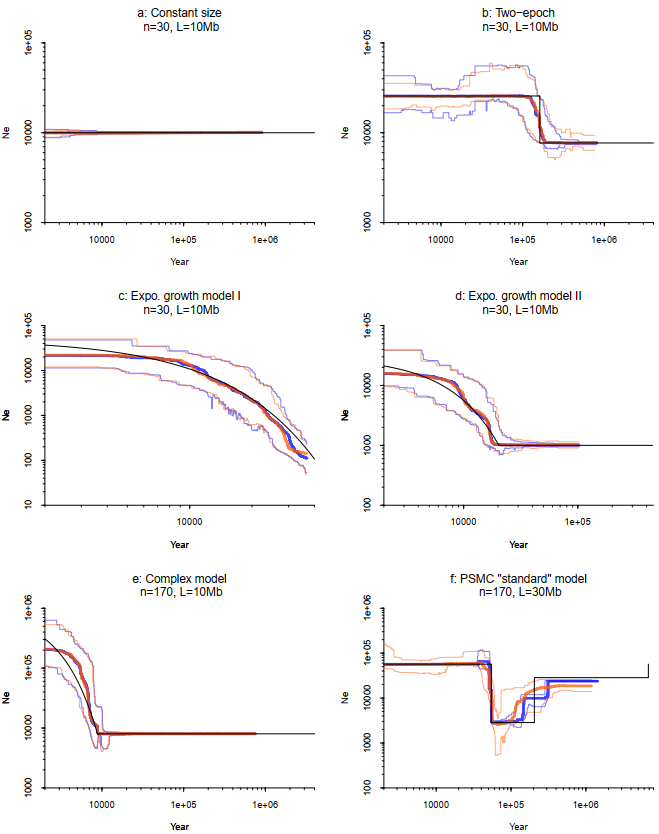
 **Fig. S1: Demographic inference comparison of Stairway Plot 2 with folded or unfolded SFS using the average SFS from 200 simulations**. Each simulation simulated *n* sequences with *L* in length each. A total of 200 independent simulations were conducted.a: assuming the constant size model^1^. b: assuming the two-epoch model^1^. c: assuming an exponential growth model^1^. d: assuming another exponential growth model^1^. e: assuming a fast growth model^1^. f: assuming the standard model used in the PSMC paper^2^. Black line: true model. Thick orange line: median of 200 estimations with folded SFS. Thin orange lines: 5% and 95% of 200 estimations with folded SFS. Thick blue line: median of 200 estimations with unfolded SFS. Thin blue lines: 5% and 95% of 200 estimations with unfolded SFS.

**A**
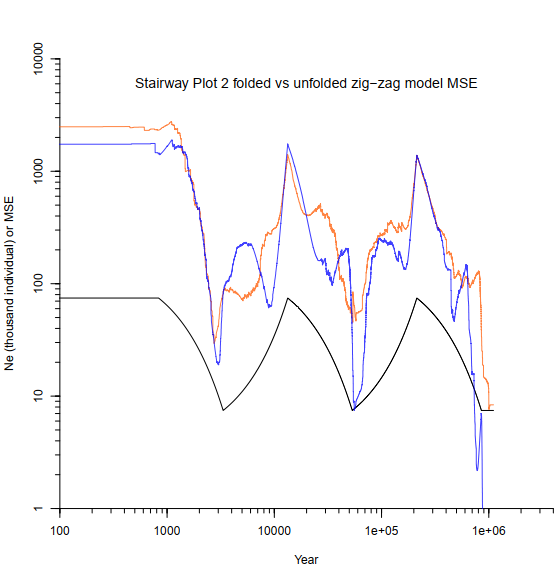


**B**


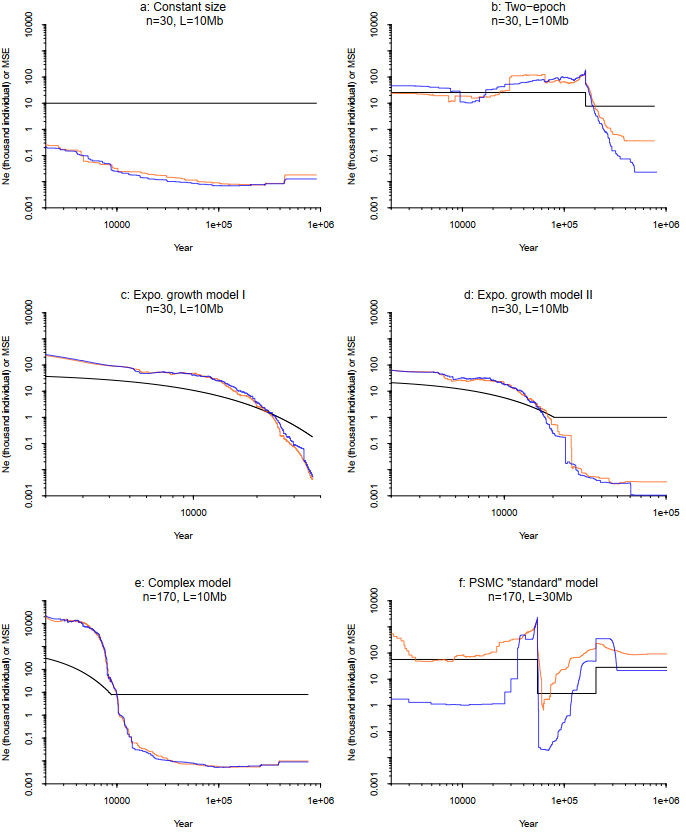


**Fig. S2**: Comparison of mean squared errors (MSEs) of 200 subsample estimations with either folded or unfolded SFSs calculated with Staiway Plot 2. **A**: The 200 subsample estmiations are the same ones used to produce the final estmiations and 95% CI estimations in Fig. 1A. **B**: The 200 subsample estmiations are the same ones used to produce the final estmiations and 95% CI estimations in Supplementary Fig. 1. Black line: true model. Orange line: MSE of the 200 subsample estimations with the folded SFS. Blue line: MSE of the 200 subsample estimations with the unfolded SFS.


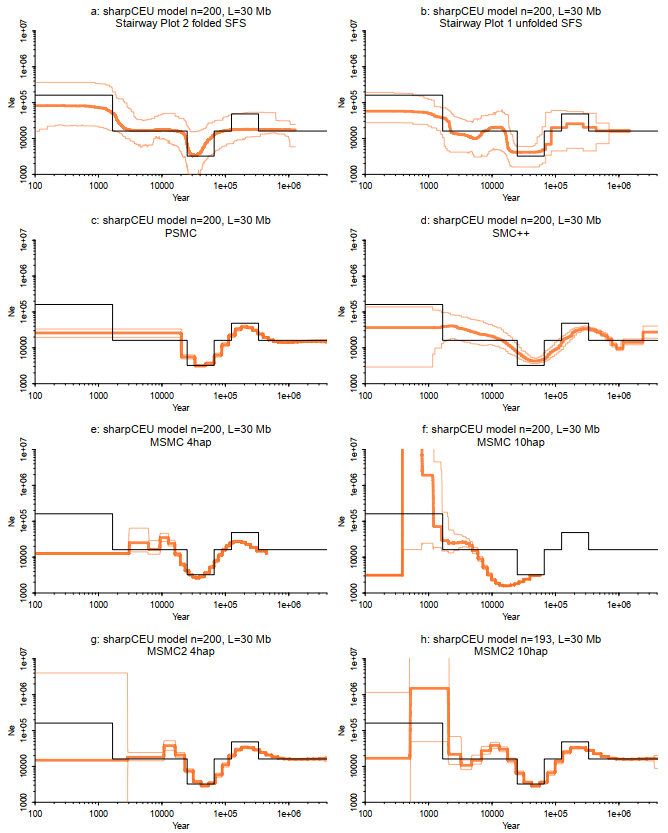
 **Fig. S3: Demographic inference comparison of Stairway Plot 2, Stairway Plot 1, PSMC, SMC++, MSMC and MSMC2 assuming the sharpCEU model**^3^. Each simulation simulated *n* sequences with *L* in length each. A total of 200 independent simulations were conducted. Stairway Plot 2, Stairway Plot 1, PSMC, SMC++, MSMC and MSMC2 were applied to the same sample of 200 sequences for each simulation. MSMC and MSMC2 group samples with every 4 haplotypes (4hap) or every 10 haplotypes (10hap). n=193 in h because MSMC2 failed to obtain the final estimation for 7 samples. Black line: true model. Thick orange line: median of 200 estimations. Thin orange lines: 5% and 95% of 200 estimations.


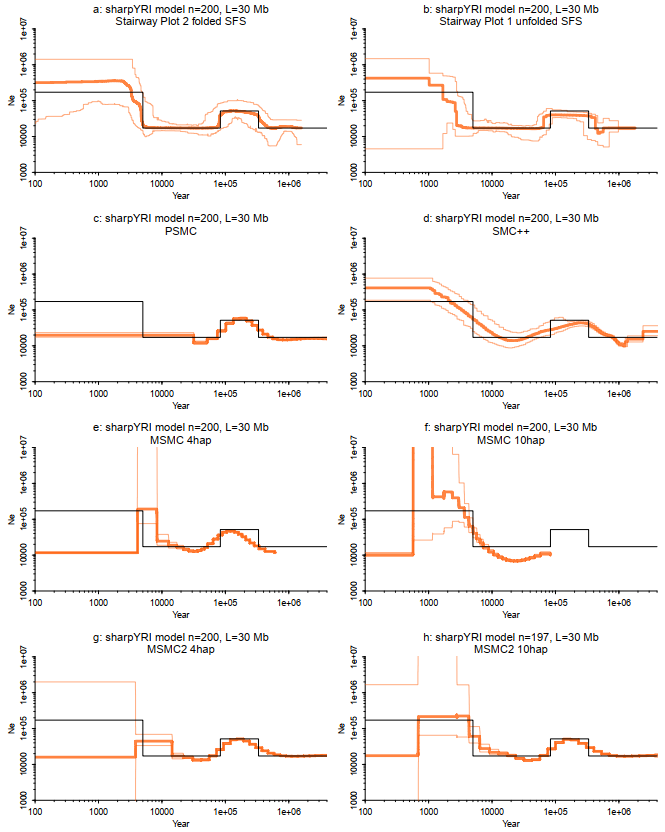


**Fig. S4: Demographic inference comparison of Stairway Plot 2, Stairway Plot 1, PSMC, SMC++, MSMC and MSMC2assuming the sharpYRI model**^3^. Each simulation simulated *n* sequences with *L* in length each. A total of 200 independent simulations were conducted. Stairway Plot 2, Stairway Plot 1, PSMC, SMC++, MSMC and MSMC2 were applied to the same sample of 200 sequences for each simulation. MSMC and MSMC2 group samples with every 4 haplotypes (4hap) or every 10 haplotypes (10hap). n=197 in h because MSMC2 failed to obtain the final estimation for 3 samples. Black line: true model. Thick orange line: median of 200 estimations. Thin orange lines: 5% and 95% of 200 estimations.

**Reference**

1. Liu, X. & Fu, Y.-X. Exploring population size changes using SNP frequency spectra. *Nat. Genet.* **47**, 555–559 (2015).

2. Li, H. & Durbin, R. Inference of human population history from individual whole-genome sequences. *Nature* **475**, 493–496 (2011).

3. Schiffels, S. & Durbin, R. Inferring human population size and separation history from multiple genome sequences. *Nat. Genet.* **46**, 919–925 (2014).
