# Supplementary material for: Stairway Plot 2: demographic history inference with folded SNP frequency spectra
Source: Genome Biol. 2020 Nov 17;21:280. doi: 10.1186/s13059-020-02196-9 (PMC7670622; doi:10.1186/s13059-020-02196-9)
Supplement: Supplementary file 2 — Additional file 2. Review History. [file 13059_2020_2196_MOESM2_ESM.docx]

**Review History**

**First round of review**

**Reviewer 1**

This method article presents a method named stairway plot 2 aiming at inferring demography from folded SFS spectra. The method seems promising and the article provides convincing evidence of its efficiency.
However, while the method is compared with existing methods, I was surprised that it was not compared with MSMC, which is supposed to perform better for the estimation of recent history. I think that such comparison would be quite helpful. A comparison with popsizeABC (Boitard et al 2016 PLoS Genet 12(3): e1005877), if possible, would also be helpful.
Moreover, the main drawback of the method is that it seems difficult to run, I have tried myself to run it and when I run the first example on a unix system:
java -cp stairway_plot_es Stairbuilder two-epoch.blueprint
I got the following error message.
Error: Could not find or load main class -cp
I would, therefore, definitely suggest the authors to develop a friendly interface (e.g. a Graphical User Interface) to run their program.

**Reviewer 2**

This manuscript presents an improved and extended version of the authors' previous stairway plot demographic estimation method, which, in the spirit of the skyline plots of phylogenetics, reconstructs a piecewise-constant estimate of population size across time but uses SFS. The new stairway plot 2 can handle folded SFS and features improved model selection and computational complexity.

I found this paper to be well written and thought its results were well-articulated and interesting. However, I do have some questions and comments (listed below), which hopefully will improve the completeness and clarity of the script. My main suggestions centre around providing more explanation or validation for some of the technical points made and including a few extra details/clearer comparisons for readers who might not be familiar with stairway plot 1.

Comments
a.     Fig 1: why do the stairway plot 2 folded figures in A and B look different? I assume it has to do with the single estimation used for comparison in B but would be nice for readers to have a little more clarification.
b.     Fig 1: as in the original stairway plot paper the benefit of these approaches over PSMC/SMC++ is performance in the more recent past vs the ancient past. Given the log x axis (which is completely reasonable) this ancient past looks quite small in comparison to the recent past and so visually the PSMC/SMC++ might appear quite bad before inspecting more closely. Could a line or two be included just to reiterate the nuances among the time periods over which these methods outperform one another?
c.     P2 L32-37: it might be worth mentioning or referencing that this is a necessary condition for statistical identifiability?
d.     P2 L47-9: an elaboration on this loss of information and its impacts would be most useful especially since the major point of stairway plot 2 is the different SFS. In general, more information on the impacts (both to methodology and inference) of the different spectra would be helpful.
e.     P2 L55-57: perhaps include a reference to recent work (e.g. Parag & Donnelly, Syst Biol, 2020) that explicitly supports this claim for piecewise-constant models and their need for proper model selection.
f.     P4 L25-8 & P9 L25-30: a supervised learning approach to model selection is suggested. Why aren't standard criteria (AIC/BIC etc) sufficient here given that the number of breakpoints directly controls parameter dimension?
g.     P5 L32-4: can a table or some data be provided to confirm the 10x speed-up maybe in the supplement? This is only necessary if the authors believe the improved computational complexity is a major selling point of stairway plot 2.
h.     P7 L22-4 & P8 L9-12: the main point that is not clear is why does it appear so easy to remove these challenging steps, but the multinomial likelihood appears quite similar to stairway plot 1? Basically, the distinction to inference is not as obvious to readers as it maybe can be (also see comment d).
i.     Could some more methods details including some of those inherited from stairway plot 1 be included briefly in the supplement for completion, given that this is a Methods paper? Just a suggestion for readers that won't be as familiar.

**Authors’ response to reviewers**

Reviewer #1

This method article presents a method named stairway plot 2 aiming at inferring demography from folded SFS spectra. The method seems promising and the article provides convincing evidence of its efficiency.

Response: Thanks for the comments

However, while the method is compared with existing methods, I was surprised that it was not compared with MSMC, which is supposed to perform better for the estimation of recent history. I think that such comparison would be quite helpful. A comparison with popsizeABC (Boitard et al 2016 PLoS Genet 12(3): e1005877), if possible, would also be helpful.

Response: Thanks for the suggestion. We did not include MSMC into comparison in the previous version for that it requires phased and polarized sequence data therefore not friendly for nonmodel organisms, while we want to focus on the application to nonmodel organisms. In this revision, we have added comparison with MSMC (and MSMC2 as suggested by the editor). We have tried to run popsizeABC but failed the test run of their example files. Unfortunately, its documentation is very limited, and we did not find a way to fix the problems after several tries. Moreover, its performance seems not good based on their own figures in their paper. Therefore, we decided not to include popsizeABC in our comparison.

Moreover, the main drawback of the method is that it seems difficult to run, I have tried myself to run it and when I run the first example on a unix system:

java -cp stairway_plot_es Stairbuilder two-epoch.blueprint

I got the following error message.

Error: Could not find or load main class -cp

I would, therefore, definitely suggest the authors to develop a friendly interface (e.g. a Graphical User Interface) to run their program.

Response: Thanks for the comments and suggestions. We double checked our files and the readme documents. We found that in our readme file, which is originally a Microsoft Word document, some of the “-” in “-cp” has be automatically changed to a longer “–”. If just copy and paste the command, it causes problem with Java. We have corrected the readme file. We confirm our programs work fine under Windows and Linux operating systems. Please try one more time with the updated program at https://github.com/xiaoming-liu/stairway-plot-v2/blob/master/stairway_plot_v2.1.1.zip. I totally agree that a GUI will be good for users. But it will need some time and effort to do it. I will add it to our to-do list for the next version.

Reviewer #2

This manuscript presents an improved and extended version of the authors' previous stairway plot demographic estimation method, which, in the spirit of the skyline plots of phylogenetics, reconstructs a piecewise-constant estimate of population size across time but uses SFS. The new stairway plot 2 can handle folded SFS and features improved model selection and computational complexity.

I found this paper to be well written and thought its results were well-articulated and interesting. However, I do have some questions and comments (listed below), which hopefully will improve the completeness and clarity of the script. My main suggestions centre around providing more explanation or validation for some of the technical points made and including a few extra details/clearer comparisons for readers who might not be familiar with stairway plot 1.

Response: Thanks for your comments.

Comments

a. Fig 1: why do the stairway plot 2 folded figures in A and B look different? I assume it has to do with the single estimation used for comparison in B but would be nice for readers to have a little more clarification.

Response: Sorry for the confusion. Yes, your assumption is correct. We modified the following sentence: “We compared the performance of Stairway Plot 2 using either unfolded SFSs or folded SFSs with the same single SFS …”. In the figure legend of Fig. 1, we added the sentence: “Only one estimation for each simulated sample was used for Stairway Plot 1 and Stairway Plot 2.”

b. Fig 1: as in the original stairway plot paper the benefit of these approaches over PSMC/SMC++ is performance in the more recent past vs the ancient past. Given the log x axis (which is completely reasonable) this ancient past looks quite small in comparison to the recent past and so visually the PSMC/SMC++ might appear quite bad before inspecting more closely. Could a line or two be included just to reiterate the nuances among the time periods over which these methods outperform one another?

Response: Thanks for the suggestion. We have added the sentence “Please note that in the figures we used log-scale for both the time (x-axis) and effective population size (y-axis), which emphasizes more recent histories and smaller population sizes.” at the place we first mentioning the figures.

c. P2 L32-37: it might be worth mentioning or referencing that this is a necessary condition for statistical identifiability?

Response: Thanks for the suggestion. We modified the sentence as “For the reason of the identifiability of the demographic model (Bhaskar and Song 2014 Annals of Statistics 42: 2469-2493), the maximum number of epochs …”.

d. P2 L47-9: an elaboration on this loss of information and its impacts would be most useful especially since the major point of stairway plot 2 is the different SFS. In general, more information on the impacts (both to methodology and inference) of the different spectra would be helpful.

Response: Thanks for the suggestion. The impact will depend on the underlying demography. We added some illustrations of the impact of loss of information using mean squared error as a measure with the subsample estimations used in Fig. 1A and Supplementary Fig. 1. And the following discussion: “… due to loss of information. On the other hand, loss of information may help to mitigate model overfitting. Therefore, the impact of the loss of information can be complex and depends on the underlying demography. We can investigate the impact by comparing the mean squared error (MSE) of the estimations with folded or unfolded SFSs (Supplementary Fig. 2). For example, Supplementary Fig. 2A compared the MSE of 200 subsample estimations with either folded or unfolded SFSs used in Fig. 1A. For most of the history, especially for more ancient histories, the estimations with unfolded SFS have a similar or smaller MSE, while in some periods those with folded SFS have a smaller MSE.”

e. P2 L55-57: perhaps include a reference to recent work (e.g. Parag & Donnelly, Syst Biol, 2020) that explicitly supports this claim for piecewise-constant models and their need for proper model selection.

Response: Thanks for the suggestion. We have added Parag & Donnelly, Syst Biol, 2020 into our references.

f. P4 L25-8 & P9 L25-30: a supervised learning approach to model selection is suggested. Why aren't standard criteria (AIC/BIC etc) sufficient here given that the number of breakpoints directly controls parameter dimension?

Response: We believe part of the reason related to the pseudo-likelihood we used to calculate the model fitness. Generally speaking, pseudo-likelihood gives an over-confident estimation for the variability of the likelihood. AIC and BIC’s penalties may not be enough. The optimal amount of penalty depends on the underlying model including sample size, sequence length, etc. The training-testing strategy can partially solve this problem. But there may be better ways for model selection, and we will continue to pursue a better model selection method.

g. P5 L32-4: can a table or some data be provided to confirm the 10x speed-up maybe in the supplement? This is only necessary if the authors believe the improved computational complexity is a major selling point of stairway plot 2.

Response: Thanks for the suggestion. We added three examples: “For example, on a single thread of an Intel Xeon Gold 5122 CPU @ 3.60 GHz, the time required for Stairway Plot 1 to produce the results (unfolded, 200 subsample estimations) for Fig 1B was 19,096 minutes. In contrast, only 900 minutes were required for Stairway Plot 2 (unfolded, 800 subsample estimations), that is, a 21-fold speed increase. With the same setting for producing results for Supplementary Fig. 3 and 4, Stairway Plot 1 required 15,839 and 9,619 minutes, while Stairway Plot 2 required 704 and 540 minutes: a 22-fold and 18-fold speed increase, respectively, were achieved.”

h. P7 L22-4 & P8 L9-12: the main point that is not clear is why does it appear so easy to remove these challenging steps, but the multinomial likelihood appears quite similar to stairway plot 1? Basically, the distinction to inference is not as obvious to readers as it maybe can be (also see comment d).

Response: Yes, the formula to calculate the pseudo-likelihood is indeed quite similar to that in stairway plot 1, since the theoretical framework remains the sample. Since we are modeling SFS, phasing is not a problem since Stairway Plot 1. One of the major improvements for stairway plot 2 is removing the requirement of polarizing SNPs by modeling folded SFS and better model selection by using an unsupervised learning strategy. The major challenges are 1) whether stairway plot will work with half the number of observations with folded SFS, and 2) can we make up the loss of information by improving in other aspects, such as better model selection strategy. Neither challenge has an obvious answer. The results showed that the theoretical framework works with folded SFS, which is partially contributed to the ensemble step: although each individual estimation can be coarse (fewer epochs), the ensemble estimation can be smooth and accurate. The ensemble step and the training-testing strategy we adopted requires a fast computation speed because by default 4x estimations for subsamples are needed to produce the final estimation. Then we need to recode our algorithm to improve speed to make the whole package usable in practice. I modified the sentence slightly to “By modeling folded SFSs and using an unsupervised learning strategy for model selection, it provides a more accurate inference of demographic histories and is especially suitable for nonmodel organisms …”

i. Could some more methods details including some of those inherited from stairway plot 1 be included briefly in the supplement for completion, given that this is a Methods paper? Just a suggestion for readers that won't be as familiar.

Response: Thanks for the suggestion. We have added the following paragraph to the Methods section: “Brief introduction of the stairway plot method. The flexible multi-epoch model used in the skyline plot method is implemented for the Stairway Plot (Pybus et al. 2000; Drummond et al. 2005), which divides time into a series of blocks with each block starting and ending at the exact time of a particular coalescent event in the sampled sequences. The population size is assumed to remain constant within each block and to be able to change from one block to the next. A maximum of n-1 time blocks can be defined given a sample of n DNA sequences, with block k corresponding to the k-coalescent time. Those n-1 time blocks can be approximated to any demographic history. The Stairway Plot estimates a series of θ_k,k=2,3,…,n, maximizing the likelihood of the observed SFS. θk = 4Nkµ, where Nk is the effective size of the population during time block k, and µ is the mutation rate per bp per generation. In practice, adjacent blocks of time can be fused into one block to reduce the parameters to be estimated. More details of the algorithm can be found in Liu and Fu (2015).”

**Second round of review**

**Reviewer 1**

The authors have added the comparison with MSMC that I requested. I could also this time run their program on my computer. I do not have any further comment and I consider that the paper can be accepted for publication.

**Reviewer 2**

The authors have responded clearly and convincingly to all of my comments. I have no further concerns and especially appreciate the responses to d and h, which clarified the inferential power of this method. Should the authors choose they may include some more of their response to h in the text to provide more intuition to the reader but this is purely optional.

**Authors’ response to reviewers**

Response: Thanks. we have add the following to the manuscript text from my response to reviewer #2 point h: “One of the major improvements for Stairway Plot 2 is removing the requirement of polarizing SNPs by modeling folded SFS, and better model selection by using an unsupervised learning strategy. The major challenges are 1) whether the Stairway Plot framework will work with half the number of observations with folded SFS, and 2) whether the loss of information can be compensated by better model selection strategy. The results showed that the Stairway Plot framework works with folded SFS and performs well with the new model selection strategy. This is partially contributed to the ensemble step: although each individual estimation can be coarse (fewer epochs), the ensemble estimation can be smooth and accurate.”
